# Epigenetic change in kidney tumor: downregulation of histone acetyltransferase MYST1 in human renal cell carcinoma

**DOI:** 10.1186/1756-9966-32-8

**Published:** 2013-02-09

**Authors:** Yong Wang, Rui Zhang, Donglu Wu, Zhihua Lu, Wentao Sun, Yong Cai, Chunxi Wang, Jingji Jin

**Affiliations:** 1 Urology Department, The First Clinical Hospital, Jilin University, Changchun City, Jilin 130021, China; 2 Urology Department, Jilin province People’s Hospital, , Changchun City, Jilin 130021, China; 3 College of Life Science, Jilin University, Changchun City, Jilin 130012, China; 4 Graduate School of Jilin University, Changchun City, Jilin 130012, China

**Keywords:** Renal cell carcinoma, hMOF, Carbonate anhydrase IX

## Abstract

**Background:**

MYST1 (also known as hMOF), a member of the MYST family of histone acetyltransferases (HATs) as an epigenetic mark of active genes, is mainly responsible for histone H4K16 acetylation in the cells. Recent studies have shown that the abnormal gene expression of hMOF is involved in certain primary cancers. Here we examined the involvement of hMOF expression and histone H4K16 acetylation in primary renal cell carcinoma (RCC). Simultaneously, we investigated the correlation between the expression of hMOF and clear cell RCC (ccRCC) biomarker carbohydrase IX (CA9) in RCC.

**Materials and methods:**

The frozen RCC tissues and RCC cell lines as materials, the reverse transcription polymerase chain reaction (RT-PCR), western blotting and immunohistochemical staining approaches were used.

**Results:**

RT-PCR results indicate that hMOF gene expression levels frequently downregulated in 90.5% of patients (19/21) with RCC. The reduction of hMOF protein in both RCC tissues and RCC cell lines is tightly correlated with acetylation of histone H4K16. In addition, overexpression of CA9 was detected in 100% of ccRCC patients (21/21). However, transient transfection of hMOF in ccRCC 786–0 cells did not affect both the gene and protein expression of CA9.

**Conclusion:**

hMOF as an acetyltransferase of H4K16 might be involved in the pathogenesis of kidney cancer, and this epigenetic changes might be a new CA9-independent RCC diagnostic maker.

## Introduction

Changes of chromatin structure are mainly regulated by epigenetic regulations including ATP-dependent remodeling of nucleosomes, the incorporation of variants histones into nucleosomes and posttranslational modifications of histones [1]. Post-translational modifications of the N-terminal tails of histones include acetylation, methylation, phosphorylation, ubiquitination, sumoylation, and ADP-ribosylation [2,3]. Histone acetylation as one of the best characterized epigenetic modifications is controlled by histone acetyltransferases (HATs) and histone deacetylases (HDAC). The balance between histone acetylation and deacetylation serves as a key epigenetic mechanism for gene expression, DNA repair, developmental processes and tumorigenesis [4-6]. Thus, any reason to make this imbalance can lead to abnormal cell function, even cancer.

MYST1 (also known as hMOF), is the human ortholog of the *Drosophila* MOF protein containing chromodomain and acetyl-CoA binding motif which is one of the key components of the dosage compensation complex (DCC) or the male specific lethal (*d*MSL) complex [7]. Recent biochemical purifications revealed that hMOF forms at least two distinct multi-protein complexes in mammalian cells. One complex is the evolutionary conserved human MSL complex which is responsible for the majority of histone H4 acetylation at lysine 16 [8,9]. The other hMOF-containing complex is the human non-specific lethal (NSL) complex which is recently characterized by Cai Y et al. [10]. hNSL complex can also acetylate histone H4 at lysine 5 and 8 on the recombinant polynucleosomes with the exception of histone H4K16. Although the functions of hMSL and hNSL complexes in human cells are not very clear, both complexes can acetylate histone H4 at lysine 16, suggesting the importance of acetylation of H4K16 in cells. Except for acetylation of H4K16, NSL complex was found to be able to acetylate the tumor suppressor protein p53, and this acetylation is able to affect the behavior of p53 in response to DNA damage [11]. It has been reported that depletion of hMOF in human cells leads to genomic instability, spontaneous chromosomal aberrations, cell cycle defects, reduced transcription of certain genes, and defective DNA damage repair and early embryonic lethality [4-7]. This suggests a critical role for hMOF in fundamental processes such as gene transcription, cell proliferation, differentiation and DNA repair response. It is worth mentioning that depletion of hMOF also leads to global reduction of histone H4K16 acetylation in human cells [8,12]. However, recent studies suggest that the global modification status of H4K16Ac is also affected by Gcn-5-containing HAT and SIRT-LSD1 HDAC complexes [13,14], indicating hMOF might not be the only HAT fulfilling acetylation of H4K16 in cells. Although the role of histone H4K16 acetylation in transcription regulation is not completely understood, loss of H4K16 acetylation has been found in certain cancers. Pfister et al. [15] found that frequent downregulation of hMOF in large series of primary breast carcinomas and medulloblastomas and hMOF protein expression tightly correlated with acetylation of H4K16 in both cancers. In addition, analysis of the tissue microarray slides revealed low or absent histone H4K16 acetylation in majority of breast cancer tissues [16].

Renal cell carcinoma (RCC) is one of the most common genitourinary malignancies, accounting for about 3% of all cancers worldwide [17]. With the improved imaging diagnostic technology, more RCC cases have been diagnosed at an early stage. However, there is a considerable number of RCC patients at the time of diagnosis has been transferred [18]. Research efforts have found various biomarkers of diagnostic and prognostic of RCC such as hypoxia-induced factor 1alpha (HIF1α), vascular endothelial growth factor (VEGF), and carbonic anhydrase IX (CA9), but they are not specific and sensitive enough to accurately predict the survival of RCC patients [19-21]. Recent studies indicate that epigenetic alterations play an important role in carcinogenesis, and global histone modifications as predictors of cancer recurrence in various tumor entities has begun to study. Patients with RCC have been found that total acetylation levels of histone H3 were inversely correlated with pT-stage, distant metastasis, Fuhrman grading and RCC progression, whereas total histone H4Ac deacetylation was correlated with pT-stage and grading [22]. All the above observations strongly suggest that histone modifications might be involved in the development and progression of RCC. However, it is not clear which particular enzyme or specific modified lysine residue is responsible for tumorigenesis in RCC. This study aims to assess hMOF expression and its corresponding acetylation of histone H4K16 in the RCC via qRT-PCR, western blotting and immunohistochemistry. Simultaneously, we also investigated the correlation between the expression of hMOF and CA9.

## Materials and methods

### Materials

Anti-H4K16 (Cat# H9164) polyclonal antibody was purchased from Sigma. Anti-MYST1 (Cat# A300-992A) was obtained from Bethyl Laboratories. Anti-CA9 (Cat# sc-25599) was from Santa Cruz Biotechnology. Anti-GAPDH and anti-hMOF rabbit polyclonal antibodies were raised against bacterially expressed proteins (Jilin University).

### Tissue collection

Human paired clinical RCC tissues and matched adjacent tissues were collected from patients with primary RCC between March 2011 and May 2012, who underwent kidney tumor radical surgery at the First Hospital of Jilin University. The study was approved by the Ethics Committee of the First Hospital of Jilin University and all patients gave informed consent. All removed tissues during the surgery were frozen immediately in liquid nitrogen and then stored at −80°C. Patient medical records including tumor staging, pathological diagnosis, and surgical records were reviewed. The pathologic diagnosis of the resected tumors was based on the American Joint Committee on Cancer [23]. All patients did not receive chemotherapy or radiotherapy before surgery.

### Cell culture and maintenance

Human embryonic kidney cell line HEK293T, human clear cell renal cell carcinoma (ccRCC) cell lines 786–0 (TCHu3) and MN/CA9 positive human renal cell carcinoma cell line OS-RC-2 (TCHu40) were obtained from Cell Resource Center of Shanghai Institute of Life Science, Chinese Academy of Science. Cells were cultured in Dulbecco’s Modified Eagle’s Medium (DMEM, Sigma) with 5% glucose and 10% fetal bovine serum, 100 U/mL penicillin, 100 mg/mL streptomycin in 10 cm dishes at 37°C in a humidified atmosphere of 5% CO2. Cultured cells were harvested from 1 well of 6-well plate and lysed using ice-cold RIPA lysis buffer (50 mM Tris HCl (pH7.4), 150 mM NaCl, 1% Nonidet P-40, 0.25% Na-deoxycholate, 1 mM EDTA and protease inhibitor cocktail). Following centrifugation at 12,000 × g for 15 min at 4°C, total proteins in resulting supernatant was quantified using the Bradford assay following the manufacturer’s instruction (BioRad).

### Western blotting

Aliquot of whole cell extract from cultured cells was mixed with 4xSDS sample buffer (0.25 M Tris–HCl pH 6.8, 8% SDS, 30% Glycerol, 0.02% Bromophenol Blue containing 10% BME). Denatured proteins were separated by SDS polyacrylamide gel (SDS-PAGE) and specific proteins were analyzed by western blotting. 200 mg of kidney tissue samples were homogenized with liquid nitrogen and solubilized in 200 μl cold PBS containing 1.0% Nonidet P-40, 0.5% Na- deoxycholate, 0.1% SDS, 0.05 mM PMSF and protease inhibitor cocktail. The homogenate was swirled and kept on ice for 30 minutes. Whole cell extracts were prepared by sonication (SCIENTZ-IID, China) for 10 seconds with 50% duty cycle and centrifugation at 12,000 rpm for 15 min. Spectrophotometer used to measure protein concentrations in a solution using a Bradford assay kit. Equal total amounts of denatured proteins were separated by SDS-PAGE. Specific proteins were detected by immunoblotting using hMOF, H4K16Ac, CA9 and GAPDH polyclonal antibodies. Immunoblotted proteins were visualized using the chemiluminescent detection system (PierceTechnology).

### Reverse transcription PCR (RT-PCR)

Cells were harvested from 1 well of a 6-well plate and total RNA was isolated using TRIzol® LS Reagent (Invitrogen). Total RNA from kidney tissues (normal/adjacent or tumor) were also isolated using TRIzol® LS Reagent. 1 μ g of RNA from each sample was used as a template to produce cDNA with PrimeScript 1st Strand cDNA Synthesis Kit (TAKARA). hMOF, CA9 and GAPDH mRNA levels were analyzed by Polymerase chain reaction (PCR) with C1000™ Thermal Cycler (BIO-RAD) and quantitative real time PCR with Real Time PCR Detector Chromo 4 (BIO-RAD). All PCR reactions were finished under following program: initial denaturation step was 95°C for 3 min, followed by 35 cycles of denaturation at 95°C for 30 seconds, annealing at 60°C for 30 seconds and extension at 72°C for 30 seconds. The primer sets used for PCR were as follows: GAPDH, 5^′^-ATCACTGCCACCCAGAAGAC-3^′^ (forward) and 5^′^-ATGAGGTCCACCACCCTGTT-3^′^ (reverse), yielding a 460 bp product; CA9, 5^′^–GCAGGAGGATTCCCCCTTG-3 (Forward) and 5^′^-GGAGCCTCAACAGTAGGTAGAT-3^′^ (Reverse), yielding a 185 bp product; hMOF, 5^′^-GGCTGGACGAGTGGGTAGACAA-3^′^ (forward) and 5^′^-TGGTGATCGCCTCATGCTCCTT-3^′^ (reverse), yielding a 227 bp product.

### Immunohistochemical staining

Formalin-fixed and paraffin-embedded clear cell renal carcinoma tissue blocks were from the The First Clinical Hospital of Jilin University. Tissue blocks were sectioned and deparaffinized in xylene and rehydrated through a graded ethanol series. Tissue slides were then subjected to antigen retrieval by boiling in 0.01 M sodium citrate buffer (pH 6) in a microwave oven for 10 min. Endogenous peroxidase was blocked by incubation for 10 min in 3% hydrogen peroxide in methanol. Finally, the reactions were detected using the DAB detection kit (Dako). Anti-MYST1 and acetylated H4K16 polyclonal antibodies were used at a 1:500 dilution. MYST1 protein expression status and the histone H4K16 acetylation levels were estimated in a four-step scale (none, weak, moderate, strong). The determination criteria are shown below: score 0 = none, no staining or nuclear staining <10% of tumor cells; score 1 = weak, partial or weak complete nuclear staining >10% of tumor cells; score 2 = moderate complete nuclear staining >10% of tumor cells; score 3 = strong and complete nuclear staining in >10% of tumor cells [24].

### Transient transfection

Human embryonic kidney (HEK) 293T cells, renal cell carcinomas 786–0 and OS-RC-2 cells were cultured in 6 well tissue culture plates (~2 × 10^5^ cells/well) in DMEM containing 10% fetal bovine serum and antibiotics. The cells were transiently transfected with 0.25~2 μg of hMOF cDNAs using polyethylenimine (PEI). At 48 hrs post-transfection, cells were harvested and lysed for immunoblot and RT-PCR analysis.

### Statistical analysis

The expression difference of genes and proteins between ccRCC and normal tissues were statistically analyzed. Statistical analysis was completed with SPSS 17.0 (SPSS, Inc., Chicago IL). Statistical comparisons were analyzed using the student’s *t*-test. Values of *P* < 0.05 were considered to be statistically significant.

## Results

### Downregulation of hMOF mRNA in primary renal cell carcinoma tissues

**Figure 1:**
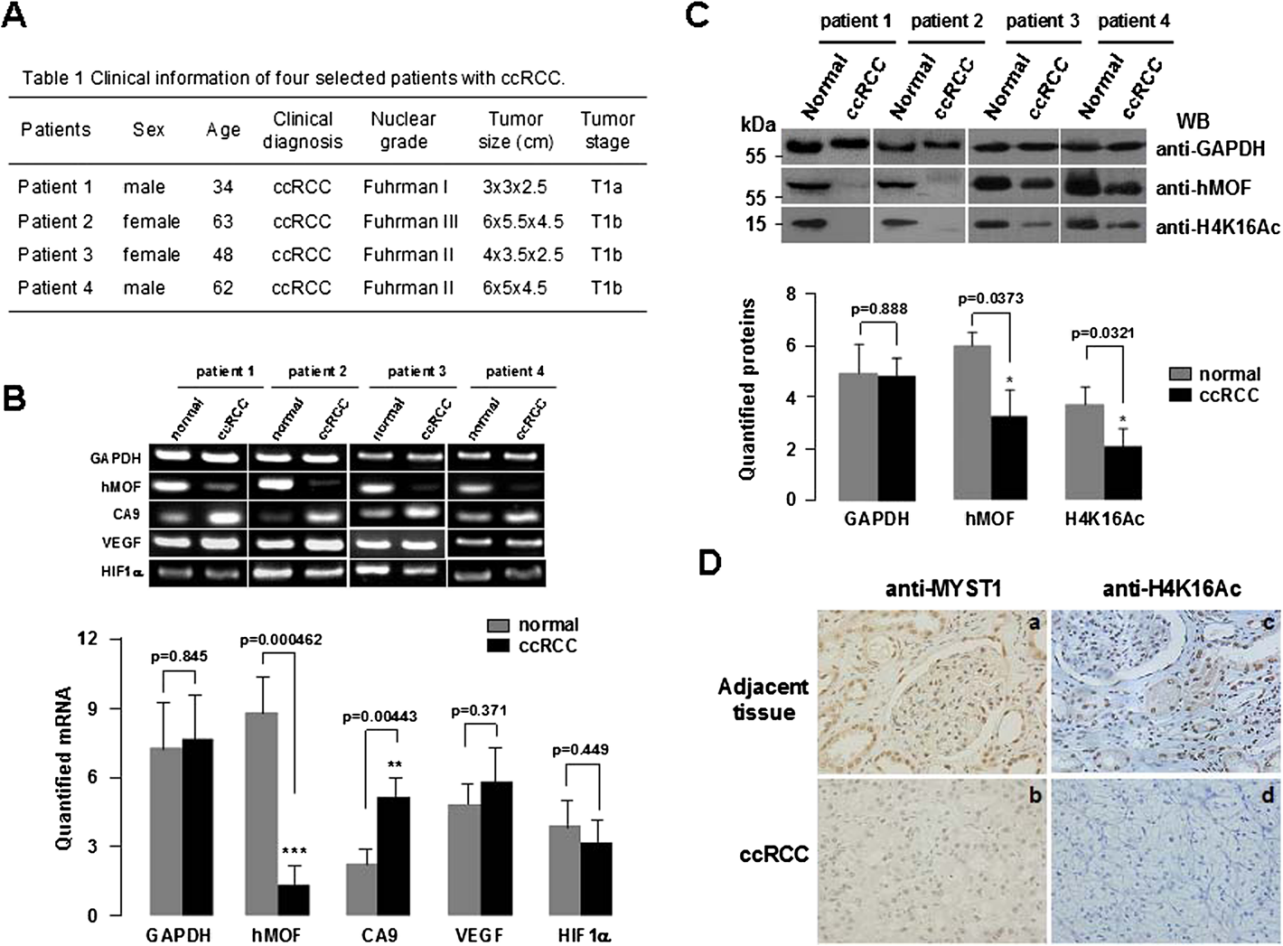
**hMOF is downregulated in human ccRCC. A.** Clinical informations of four newly diagnosed patients with ccRCC. **B. **hMOF mRNA levels are dropped down in 4 random cases of ccRCC tissues. Total RNA from tissue was isolated using trizol. mRNA levels of hMOF, CA9, VEGF and HIF1α in paired human clinical ccRCC and adjacent kidney tissue was analyzed by RT-PCR (upper panel). mRNA levels were quantified by densitometry using Quantity One Basic software (Bio- Rad) (lower panel). **C.** Total hMOF protein expression and the acetylation of histone H4K16 levels are decreased in selected ccRCC tumor tissue. Aliquots of whole cell extracts from four selected ccRCC tumor samples and its corresponding adjacent tissues were subjected to SDS-PAGE in 12% gels, and proteins were detected by western blotting with indicated antibodies (upper panel). Western blot images were quantified using Quantity One software (Bio-Rad) (lower panel). The significant difference is expressed as *p<0.05, **p<0.01, ***p<0.001. **D.** An example of immunostaining for hMOF and H4K16Ac in ccRCC. hMOF expression status in adjacent renal tissue (**a**) and in ccRCC (**b**) were visualized by immunohistochemical staning with anti-MYST1 antibody. Acetylation levels of modified histone H4K16 was immunostained by acetylation-specific antibody in adjacent renal tissue (**c**) and in ccRCC (**d**).

**Figure 2:**
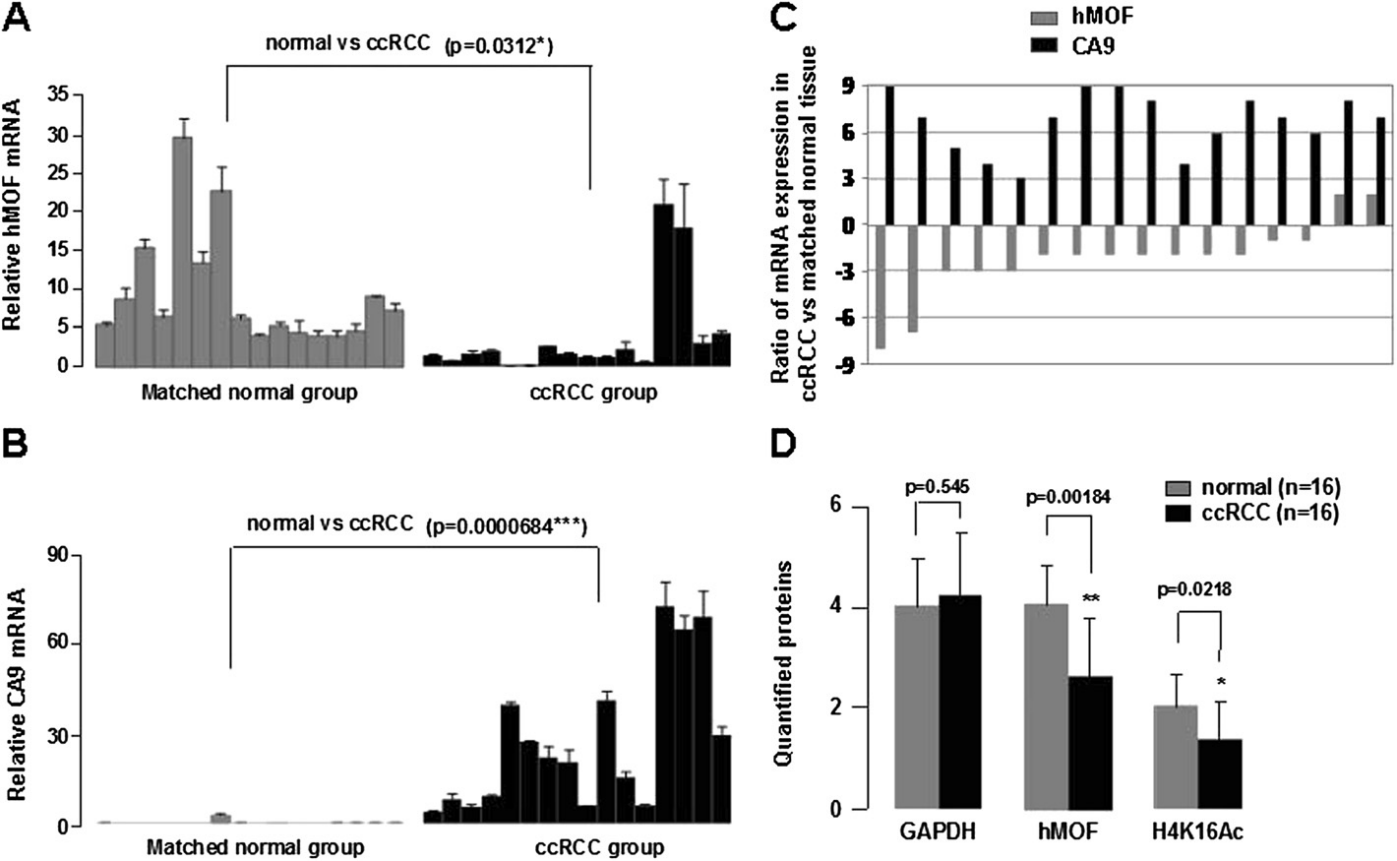
**Downregulation of hMOF is accompanied by increased CA9 in ccRCC. A-B. **Relative mRNA expression levels of hMOF and CA9 in ccRCC. Total RNA was isolated from sixteen paired clinical ccRCC and adjacent kidney tissues. Relative mRNA expression levels of hMOF and CA9 were analized by quantitative RT-PCR. Error bars represent the standard error of the mean of 3 independent experiments. Student’s *t*-test was performed to compare the difference between ccRCC and normal tissues. **C. **Expression patterns of hMOF and CA9 mRNAs in ccRCC and its corresponding adjacent kidney tissues. Expression is displayed as a ratio of expression of hMOF or CA9 gene in ccRCC versus matched normal tissues. Each bar is the log2 value of the ratio of hMOF or CA9 expression levels between ccRCC and matched normal tissues from the same patients. Bar value >1 represents >2-fold increased, whereas bar value <−1, represents >2-fold decreased. **D. **Protein expression levels of hMOF and its H4K16Ac status in ccRCC. Aliquots of whole cell extracts from sixteen selected ccRCC tumor samples and its corresponding adjacent tissues were analyzed by western blotting. The blots were then scanned and quantified with Quantity One software. The significant difference is expressed as *p<0.05, **p<0.01.

**Figure 3:**
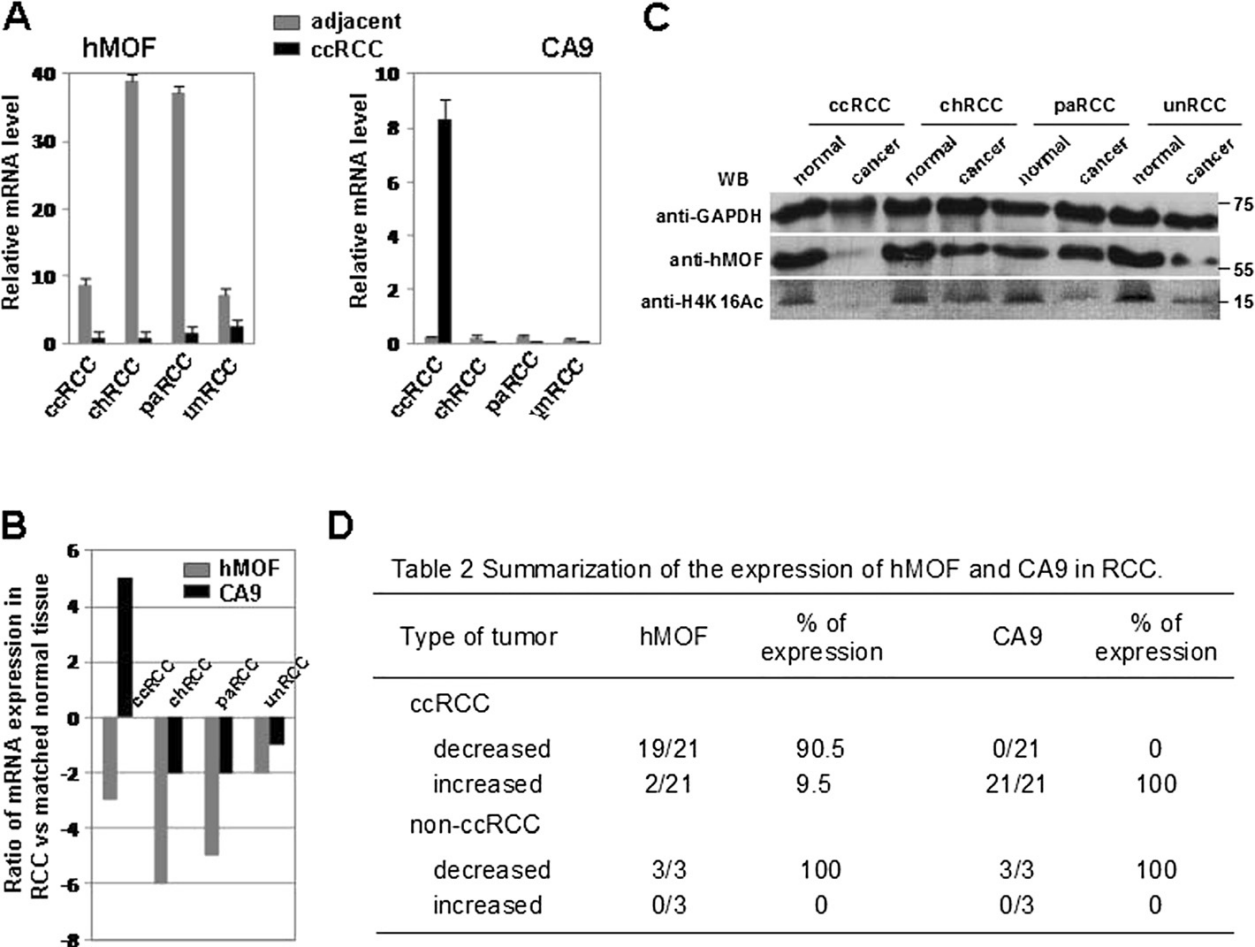
**hMOF is downregulated in different pathological diagnosis of human kidney cancer. A. **Relative mRNA expression levels of hMOF in different type of kidney cancer. Total RNA was isolated from four paired pathological diagnosed ccRCC, chRCC, paRCC, unclassified RCC, respectively and matched normal/adjacent kidney tissues. Relative mRNA expression levels of hMOF and CA9 were analyzed by qRT-PCR. Error bars represent the standard error of the mean of 3 independent experiments. **B. **Log2 ratio of hMOF and CA9 mRNA expression in four different types of human kidney cancer. Ratio of mRNA expression is displayed as a ratio of expression of hMOF or CA9 gene in ccRCC versus matched normal tissues. **C. **Analysis of werstern blotting. Equivalent total protein amount of whole cell extracts from four different pathological diagnosed kidney cancers (ccRCC, chRCC, paRCC and unRCC) and its corresponding normal/adjacent tissues were subjected to SDS-PAGE in 12% gels, and proteins were detected by western blotting with indicated antibodies. **D. **Summarization of hMOF and CA9 expression in RCC. Total cases of ccRCC (21) include four initial selected ccRCC (data not shown), sixteen additional ccRCC and one case used in comparing experiment.

In order to know whether the hMOF is involved in the pathogenesis of primary RCC or not, we first examined the mRNA levels of hMOF and other hypoxia signature genes including CA9, VEGF and HIF1α in 4 random cases of newly diagnosed ccRCC (Figure 1A) by reverse transcription PCR (RT-PCR) and quantitative real-time PCR (qPCR). As shown in Figure 1B, the gene expression levels of hMOF were markedly decreased in all ccRCC tissues compared to matched normal tissues (p<0.001). In contrast, CA9 expression levels were significantly increased in all ccRCC tissues (p<0.01). However, no significant difference was observed in VEGF and HIF1α expression. Additional 16 paired clinical ccRCC and matched normal tissues were used to further validate the frequent downregulation of hMOF mRNA expression in primary ccRCC. Analysis of performed mRNA expression of 16 samples revealed significant (>2-fold decreased) downregulation of hMOF mRNA in 87.5% (14/16) of patients (Figure 2A and C), whereas 12.5% (2/16) of patients showed significant (>2-fold increased) upregulation of hMOF (Figure 2A and C). However, less relationship between hMOF expression and tumor size, stage and grading was detected in our limited number of cases (data not shown). To examine the gene expression status of hMOF in other types of RCC, four kidney cancer patients with pathologically daignosed ccRCC, chRCC (chromophobe RCC), paRCC (papillary RCC) and unRCC (unclassified RCC), respectively, were selected. Analysis of qRT-PCR results showed that the gene expression of hMOF significantly downregulated in all types of RCC (>2-fold) (Figure 3A and B).

### Reduction of hMOF protein in human primary renal cell carcinoma tissues

The results of RT-PCR analysis clearly show frequent downregulation of hMOF gene expression in RCC. To determine whether the reduction of hMOF mRNA expression resulted in decreasing of hMOF protein levels, western blotting and immunohistochemical staining approaches were used. As shown in Figure 1C, aliquots of whole cell extract from four paired initially selected ccRCC and matched normal tissues were analyzed by western blotting with indicated antibodies. Similar to our expected results, significant reduction of hMOF protein in ccRCC compared to those of matched normal tissues were detected (p<0.05). Simultaneously, the acetylation status of histone H4K16 was also significantly reduced or lost (p<0.05). To further confirm these results, we performed immunohistochemical staining for hMOF and histone H4K16 acetylation in the formalin fixed paraffin embedded tissue sections of same four selected ccRCC patients. The results revealed that both the hMOF protein levels and the histone H4K16 acetylation status were markedly reduced (score 1 to 2 for hMOF staining, and score 0–1 for H4K16Ac staining) in all ccRCC tissues compared to adjacent tissues. For example, the results of immunohistochemical staining for hMOF and H4K16Ac are presented in Figure 1D. Weak staining of hMOF and no staining of H4K16Ac in the ccRCC paraffin embedded tissue sections were detected. In the additional 16 paired clinical ccRCC and matched normal tissues, hMOF protein expression and H4K16Ac status were detected by western blotting. The quantified protein levels (Quantity One software) were analyzed by *t-*test. As shown in Figure 2D, hMOF protein expression levels were significantly reduced in ccRCC tissues (p<0.01), and the expression of hMOF was tightly correlated with H4K16 acetylation (p<0.05). Furthermore, in the four different pathologically diagnosed ccRCC, chRCC, paRCC and unRCC, hMOF protein expression was significantly decreased in ccRCC, chRCC and unclassified RCC, whereas less changes were detected in paRCC (Figure 3C).

### Elevation of CA9 gene expression is accompanied by frequent reduction of hMOF mRNA in ccRCC

CA9 is not expressed in healthy renal tissue but is expressed in most ccRCC through HIF1α accumulation driven by hypoxia [25]. In our study, the gene expression of CA9 was significantly increased (>2-fold) in 100% of ccRCC patients (21/21; Figure 3D) including four initial selected ccRCC, sixteen additional ccRCC (Figure 2B) and one case (Figure 3A and B) used in comparing experiment. Among these cases, reduction of hMOF mRNA expression was detected in 90.5% of cases (19/21). There were only 2 cases presenting elevation of hMOF mRNA expression in ccRCC (Figure 2A and C). However, no elevation of CA9 gene expression was detected in different pathologically diagnosed RCC including chRCC, paRCC and unRCC, although the mRNA levels of hMOF were significantly decreased in those RCC (Figure 3B).

**Figure 4:**
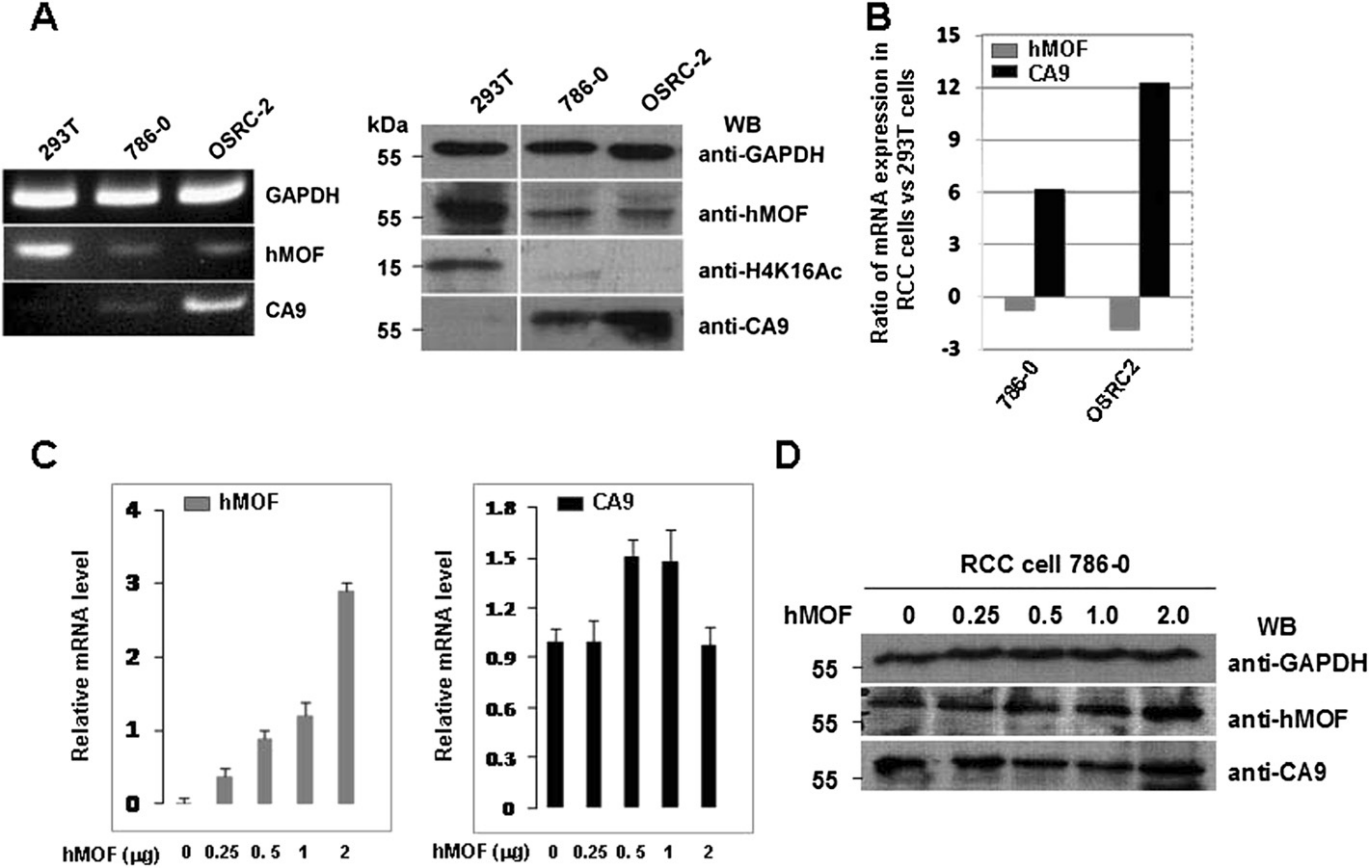
**Non-correlation between hMOF and CA9 is found in renal cell carcinoma cells. A. **hMOF protein expression was correlated with acetylation of H4K16 in RCC cell 786–0 and OSRC-2. 293T, 786–0 or OSRC-2 cells were cultured in 6-well tissue culture plates (~2x10^5^ cells/well) in DMEM medium containing 10% fetal bovine serum. Whole cell extracts were subjected to immunoblotting using indicated antibodies (right panel). 293T, 786–0 or OSRC-2 cells from 1 well of a 6 well plate were lysed and total RNA was isolated using Trizol. hMOF and CA9 gene expressions were measured by RT-PCR (left panel) and qRT-PCR (**B**). **C. **Effect of hMOF on CA9 mRNA expression levels in RCC cells. RCC 786–0 cells were cultured in 6-well tissue culture plates (~2x10^5 ^cells/well) in DMEM medium containing 10% fetal bovine serum. The cells were transfected with 0.25, 0.5, 1 and 2 μg of hMOF cDNAs. 48 hours after transfection, cells were lysed and total RNA was isolated using Trizol. Indicated gene expressions were analyzed by qRT-PCR. **D. **Effect of hMOF on CA9 protein expression in RCC cells. RCC 786–0 cells were transfected with 0.25, 0.5, 1 and 2 μg of hMOF cDNAs. 48 hours after transfection, cells were harvested and lysed in RIPA buffer. Aliquots of whole cell extracts were subjected to 12% SDS-PAGE, and specific proteins were detected by indicated antibodies.

### Non-correlation between hMOF and CA9 is found in renal cell carcinoma cells

To extend these observations and to know whether there is a correlation regulatory relationship between hMOF and CA9 in cells, we performed experiments using RCC cell 786–0 and OS-RC-2 as model. We first examined both the protein levels and mRNA expression levels of the hMOF and CA9 in 293T, 786–0 and OS-RC-2 cells. The results as shown in Figure 4A indicate the opposing gene expression patterns between hMOF and CA9 were observed. The expression of hMOF was reduced in both 786–0 and OS-RC-2 cells compared to 293T cells, and the log2 ratio changes are −0.84 and −1.9, respectively. Western blotting analysis revealed that the hMOF proteins were markedly decreased in both renal cell carcinoma cells. In addition, the reduction of hMOF proteins resulted in loss of the acetylation of histone H4K16 in RCC cells. In contrast with hMOF, the gene expression of CA9 was increased in both 786–0 (log2=6.2) and OS-RC-2 cells (log2=12.3) compared to 293T cells. To determine whether the CA9 gene expression was regulated by hMOF, renal cell carcinoma 786–0 cells were transiently transfected with 0.25 to 2 μg of hMOF cDNAs. The results are shown in Figure 4C and D, both the gene and protein expression levels of hMOF were dose-dependently increased. However, neither the gene nor protein expression of CA9 levels were affected by transient transfection RCC 786–0 cells with hMOF cDNAs.

## Discussion

The HAT hMOF belongs to the MYST (Moz-Ybf2/Sas3-Sas2-Tip60) family, and is believed to be responsible for histone H4 acetylation at lysine 16 in both *Drosophila* and human cells [7,8,12]. Abnormal expression of the hMOF and its corresponding modification of H4K16 have been found in certain primary cancer tissues. The expression behavior of hMOF in different primary cancers was observed to be different. Frequent downregulation of hMOF expression was found in primary breast cancer and medulloblastoma [15]. On the contrary, hMOF was overexpressed in non-small cell lung carcinoma tissues [26]. Regardless of what type of situation, hMOF protein expression tightly correlated with acetylation of histone H4K16. In this study, we investigated the expression of histone acetyltransferase hMOF and its corresponding H4K16 acetylation in a series of primary kidney tumor tissues by qRT-PCR, western blotting, and immunohistochemistry. The results revealed that either hMOF mRNA expression or hMOF protein expression was frequently downregulated in human RCC (19/21 cases; >90%), and hMOF protein expression was correlated with acetylation of histone H4K16 in parallel. In addition, low protein expression levels of hMOF and loss of histone H4K16 acetylation were detected in renal carcinoma cells 786–0 and OS-RC-2 compared to human embryonic kidney cell HEK293T. Together this, HAT hMOF might have an important role in primary renal cell carcinoma tumorigenesis.

CA9 is a transmembrane, zinc-containing metalloenzyme that catalyzes reversible reactions of the bicarbonate buffer system to regulate pH in hypoxic conditions [27]. Overexpression of CA9 has been shown in a wide variety of malignant cell lines and tumors [28-30]. It is worth mentioning that CA9 has been well described as a diagnostic marker for clear cell renal carcinoma (ccRCC), especially by showing high expression in metastastic ccRCC (mccRCC) [31,32]. Therefore, the inhibitor or regulatory proteins of hypoxic tumor-associated CA9 possesses the potential therapeutic possibility for those tumors in which CA9 is involved in perturbing the extra- or intra- tumoral acidification process. In our experiments, although the expression of VEGF and HIF1α which are hypoxia signature genes were not observed significant difference between ccRCC and normal tissues, overexpression of CA9 was observed in 100% of ccRCC cases and in both renal carcinoma cell lines. Interestingly, in four different diagnostic RCCs, downregulation of hMOF was detected in all types of RCCs, but the overexpression of CA9 was only presented in ccRCC, suggesting that hMOF might be a new common diagnostic marker for human different diagnostic RCC. Although frequent downregulation of hMOF and overexpression of CA9 were detected in both RCC clinical tissues and RCC cell lines, non-correlation between hMOF and CA9 was found in RCC 786–0 cells, suggesting hMOF and its corresponding modifications might be a new CA9-independent RCC diagnosis biomarker. Although large series of clinical cases and analyses of overall survival need to be investigated, the molecular mechanism linking loss of hMOF expression to renal cell carcinoma, especially mechanism of hMOF on renal cell carcinomas, will be an exciting avenue for further research.

## Conclusion

In conclusion, hMOF as an acetyltransferase of H4K16 might be involved in the pathogenesis of renal cell carcinoma, and this epigenetic change might be a new CA9-independent RCC diagnostic marker. In addition, our results suggest that a novel molecular mechanism of hMOF might serve as a lead to new therapeutics target in human renal cell carcinoma.

## Competing interests

The authors declare that they have no competing interests.

## Authors’ contributions

YW, RZ, DW and ZL carried out the experiments and data analyses. WS and CW collected the clinical samples and completed immunohistochemistry. YC and JJ drafted the manuscript. All authors read and approved the final manuscript.

## References

[B1] Jin J Cai Y Li B Conaway RC Workman JL Conaway JW Kusch T In and out: histone variant exchange in chromatin Trends Biochem Sci 2005 30 680 687 10.1016/j.tibs.2005.10.003 16257529

[B2] Berger SL The complex languige of chromatin regulation during transcription Nature 2007 447 407 412 10.1038/nature05915 17522673

[B3] Bhaumik SR Smith E Shilatifard A Covalent modifications of histones during development and disease pathogenesis Nat Struct Mol Biol 2007 14 1008 1016 10.1038/nsmb1337 17984963

[B4] Carrouzza MJ Utley RT Workman JL Cote J The divers functions of histone acetyltransferase complexes Trends Genet 2003 19 321 329 10.1016/S0168-9525(03)00115-X 12801725

[B5] Gupta A Guerin-Peyrou TG Sharma GG Park C Agarwal M Ganju RK Pandita S Choi K Sukumar S Pandita RK Ludwig T Pandita TK The mammalian ortholog of Drosophla MOF that acetylates histone H4 lysine 16 is essential for embryogenesis and oncogenesis Mol Cell Biol 2008 28 397 409 10.1128/MCB.01045-07 17967868 PMC2223300

[B6] Sharma GG So S Gupta A Kumar R Cayrou C Avvakumov N Bhadra U Pandita RK Porteus MH Chen DJ Cote J Pandita TK MOF and histone H4 acetylation at lysine 16 are critical for DNA damage response and double-strand break repair Mol Cell Biol 2010 30 3582 3595 10.1128/MCB.01476-09 20479123 PMC2897562

[B7] Rea S Xouri G Akhtar A Males absent on the first (MOF): from flies to humans Oncogene 2007 26 5385 5394 10.1038/sj.onc.1210607 17694080

[B8] Smith ER Cayrou C Huang R Lane WS Côtê J Lucchesi JC A human protein complex homologus to the Drosophila MSL complex is responsible for the majority of histone H4 acetylation at lysine 16 Mol Cell Biol 2005 25 9175 9188 10.1128/MCB.25.21.9175-9188.2005 16227571 PMC1265810

[B9] Mendjan S Taipale M Kind J Holz H Gebhardt P Schelder M Vermeulen M Buscaino A Duncan K Mueller J Wilm M Stunnenberg HG Saumweber H Akhtar A Nuclear pore components are involved in the transcriptional regulation of dosage compensation in Drosophila Mol Cell 2006 21 811 823 10.1016/j.molcel.2006.02.007 16543150

[B10] Cai Y Jin J Swanson SK Cole MD Choi SH Florens L Washburn MP Conaway JW Conaway RC Subunit composition and substrate specificity of a MOF-containing histone acetyltransferase distinct from the male-specific lethal (MSL) complex J Biol Chem 2010 285 4268 4272 10.1074/jbc.C109.087981 20018852 PMC2836030

[B11] Sykes SM Mellert HS Holbert MA Li K Marmorstein R Lane WS McMahon SB Acetylation of the p53 DNA-binding domain regulates apoptosis induction Mol Cell 2006 24 841 851 10.1016/j.molcel.2006.11.026 17189187 PMC1766330

[B12] Taiple M Rea S Richter K Vilar A Lichter P Imhof A Akhtar A hMOF histone acetyltransferase is required for histone H4 lysine 16 acetylation in mammalian cells Mol Cell Biol 2005 25 6798 6810 10.1128/MCB.25.15.6798-6810.2005 16024812 PMC1190338

[B13] Mulligan P Yang F Di Stefano L Ji JY Ouyang J Nishikawa JL Toiber D Kulkarni M Wang Q Najafi-Shoushtari SH Mostoslavsky R Gygi SP Gill G Dyson NJ Näär AM A SIRT-LSD1 Co-repressor complex regulates notch target gene expression and development Mol Cell 2011 42 689 699 10.1016/j.molcel.2011.04.020 21596603 PMC3119599

[B14] Orpinell M Fournier M Riss A Nagy Z Krebs AR Frontini M Tora L The ATAC acetyl transferase complex controls mitotic progression by targeting non-histone substrates EMBO J 2010 29 2381 2394 10.1038/emboj.2010.125 20562830 PMC2910275

[B15] Pfister S Rea S Taipale M Mendrzyk F Straub B Ittrich C Thuerigen O Sinn HP Akhtar A Lichter P The histone acetyltransferase hMOF is frequently downregulated in primary breast carcinoma and medulloblastoma and constitutes a biomarker for clinical outcome in medulloblastoma Int J Cancer 2008 122 1207 1213 18058815 10.1002/ijc.23283

[B16] Elsheikh S Green AR Rakha EA Powe DG Ahmed RA Collins HM Soria D Garibaldi JM Paish CE Ammar AA Grainge MJ Ball GR Abdelghany MK Martinez-Pomares L Heery DM Ellis IO Globle histone modifications in breast cancer correlate with tumor phenotypes, prognostic factors, and patient outcome Cancer Res 2009 69 3802 3809 10.1158/0008-5472.CAN-08-3907 19366799

[B17] Jemal A Siegel E Ward E Murray T Xu J Thun MJ Cancer stastistics CA Cancer J Clin 2007 57 43 66 10.3322/canjclin.57.1.43 17237035

[B18] Janzen NK Kim HL Figlin RA Belldegrun AS Surveillance after radical or partial nephrectomy for localized renal cell carcinoma and management of recruitment disease Urol Clin North Am 2003 30 843 852 10.1016/S0094-0143(03)00056-9 14680319

[B19] Eichelberg C Junker K Ljungberg B Moch H Diagnostic and prognostic molecular markers for renal cell carcinoma: a critical appraisal of the current state of research and clinical applicability Eur Urol 2009 55 851 863 10.1016/j.eururo.2009.01.003 19155123

[B20] Belldegrun AS Renal cell carcinoma: prognostic factors and patient selection Eur Urol Suppl 2007 6 477 483 10.1016/j.eursup.2007.01.016

[B21] Wu XR Sha JJ Liu DM Chen YH Yang GL Zhang J Xhen YY Bo JJ Huang YR High expression of p53-induced ring-h2 protein is associated with poor prognosis in clear cell renal cell carcinoma Eur J Sur Oncol 2012 39 100 106 10.1016/j.ejso.2012.10.004 23102595

[B22] Mosashvilli D Kahl P Mertens C Holzapfel S Rogenhofer S Hauser S Büttner R Von Ruecker A Müller SC Ellinger J Globle histone acetylation levels: prognostic relevance in patients with renal cell carcinoma Cancer Sci 2010 101 2664 2669 10.1111/j.1349-7006.2010.01717.x 20825416 PMC11159183

[B23] Edge SB Byrd DR Compton CC Fritz AG Greene FL Trotti A AJCC Cancer Staging Manual 2010 7 Chicago, IL: Springer

[B24] Choschzick M Oosterwijl R Muller V Woelber L Simon R Moch H Tennstedt P Overexpression of carbonic anhydrase IX (CAIX) is an independent unfavorable prognostic marker in endometrioid ovarian cancer Virchows Arch 2011 459 193 200 10.1007/s00428-011-1105-y 21691815

[B25] Tostain J Li G Gentil-Perret A Gigante M Carbonate anhydrase 9 in clear cell renal cell carcinoma: A marker for diagnosis, prognosis and treatment Eur J Cancer 2010 46 3141 3148 10.1016/j.ejca.2010.07.020 20709527

[B26] Song JS Chun SM Lee JY Kim DK Kim YH Jang SJ The histone acetyltransferase hMOF is overexpressed in non-small cell lung carcinoma Korean J Pathol 2011 45 386 396 10.4132/KoreanJPathol.2011.45.4.386

[B27] Stillebroer AB Mulders PF Boerman OC Oyen WJ Oosterwijk E Carbonic anhydrase IX in renal cell carcinoma: implications for prognosis, daignosis, and therapy Eur Urol 2010 58 75 83 10.1016/j.eururo.2010.03.015 20359812

[B28] Hussain SA Ganesan R Reynolds G Gross L Stevens A Pastorek J Murray PG Perunovic B Anwar MS Billingham L James ND Spooner D Poole CJ Rea DW Palmer DH Hypoxia-regulated carbonic anhydrase IX expression is associated with poor survival in patients with invasive breast cancer Br J Cancer 2007 96 104 109 10.1038/sj.bjc.6603530 17213826 PMC2360224

[B29] Klatte T Seligson DB Rao JY Yu H de Martino M Kawaoka K Wong SG Belldegrun AS Pantuck AJ Carbonic anhydrase IX in bladder cancer: a diagnostic, prognostic, and therapeutic molecular marker Cancer 2009 115 1448 1458 10.1002/cncr.24163 19195047

[B30] Swinson DE Jones JL Richardson D Wykoff C Turley H Pastorek J Harris AL O’Byrne KJ Carbonic anhydrase IX expression, a novel surrogate marker of tumor hypoxia, is associated with a poor prognosis in non-small-cell lung cancer J Clin Oncol 2003 21 473 482 10.1200/JCO.2003.11.132 12560438

[B31] Leibovich BC Sheinin Y Lohse CM Thompson RH Cheville JC Zavada J Kwon ED Carbonic anhydrase IX is not an independent predictor of outcome for patients with clear cell renal cell carcinoma J Clin Oncol 2007 25 4757 4764 10.1200/JCO.2007.12.1087 17947723

[B32] Liao SY Aurelio ON Jan K Zavada J Stanbridge EJ Identification of the MN/CA9 protein as a reliable diagnostic biomarker of clear cell carcinoma of the kidney Cancer Res 1997 57 2827 2831 9230182

